# Current status of developed electrocatalysts for water splitting technologies: from experimental to industrial perspective

**DOI:** 10.1186/s40580-024-00468-9

**Published:** 2025-02-06

**Authors:** Duy Thanh Tran, Phan Khanh Linh Tran, Deepanshu Malhotra, Thanh Hai Nguyen, Tran Thien An Nguyen, Nguyen Tram Anh Duong, Nam Hoon Kim, Joong Hee Lee

**Affiliations:** 1 Department of Nano Convergence Engineering, Jeonbuk National University, Jeonju, Jeonbuk, 54896 Republic of Korea; 2 Carbon Composite Research Center, Department of Polymer-Nano Science and Technology, Jeonbuk National University, Jeonju, Jeonbuk, 54896 Republic of Korea

**Keywords:** Electrocatalyst, Hydrogen evolution reaction, Oxygen evolution reaction, Water electrolysis

## Abstract

**Abstract:**

The conversion of electricity into hydrogen (H_2_) gas through electrochemical water splitting using efficient electrocatalysts has been one of the most important future technologies to create vast amounts of clean and renewable energy. Low-temperature electrolyzer systems, such as proton exchange membrane water electrolyzers, alkaline water electrolyzers, and anion exchange membrane water electrolyzers are at the forefront of current technologies. Their performance, however, generally depends on electricity costs and system efficiency, which can be significantly improved by developing high-performance electrocatalysts to enhance the kinetics of both the cathodic hydrogen evolution reaction and the anodic oxygen evolution reaction. Despite numerous active research efforts in catalyst development, the performance of water electrolysis remains insufficient for commercialization. Ongoing research into innovative electrocatalysts and an understanding of the catalytic mechanisms are critical to enhancing their activity and stability for electrolyzers. This is still a focus at academic institutes/universities and industrial R&D centers. Herein, we provide an overview of the current state and future directions of electrocatalysts and water electrolyzers for electrochemical H_2_ production. Additionally, we describe in detail the technological framework of electrocatalysts and water electrolyzers for H_2_ production as utilized by relevant global companies.

**Graphical Abstract:**

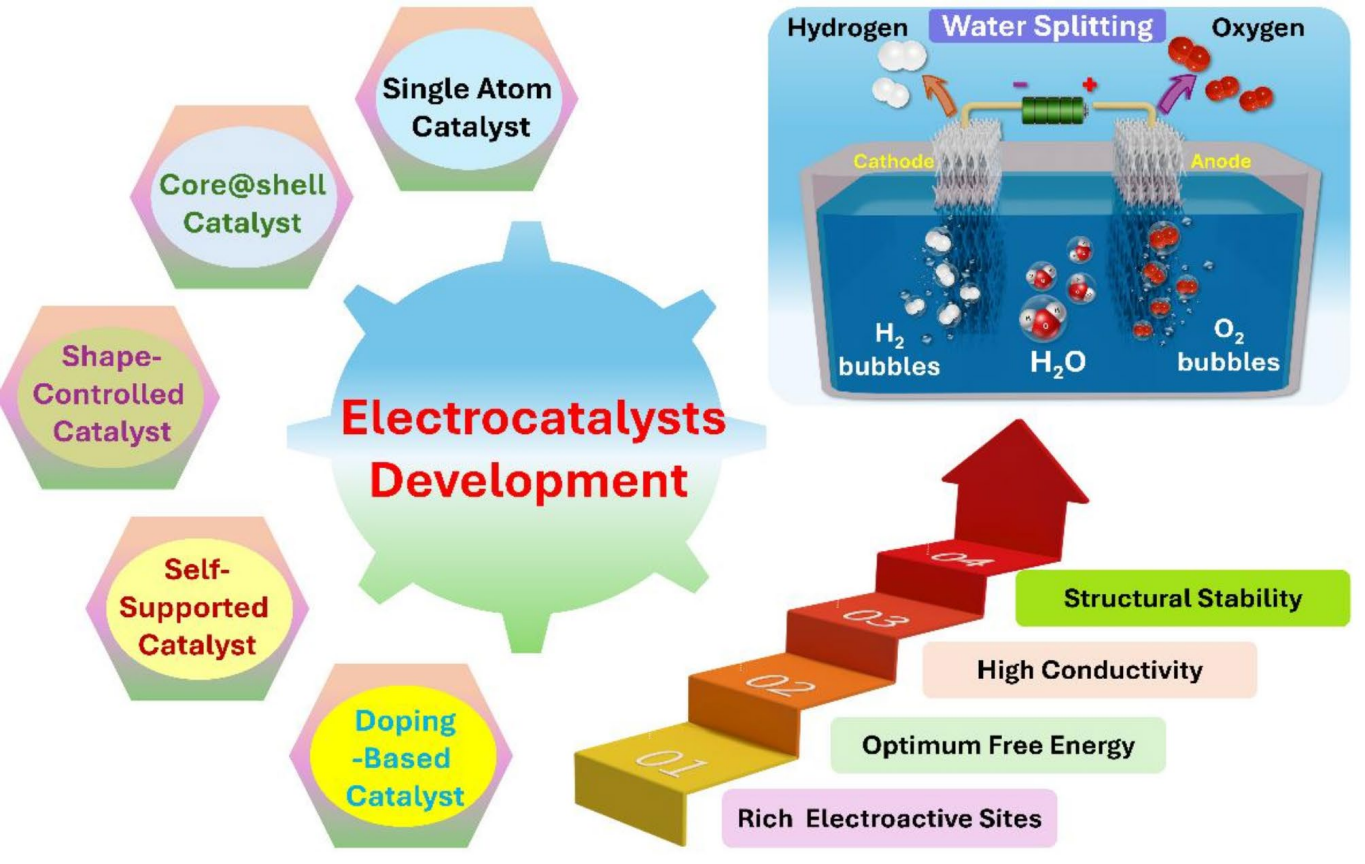

## Introduction

**Fig. 1:**
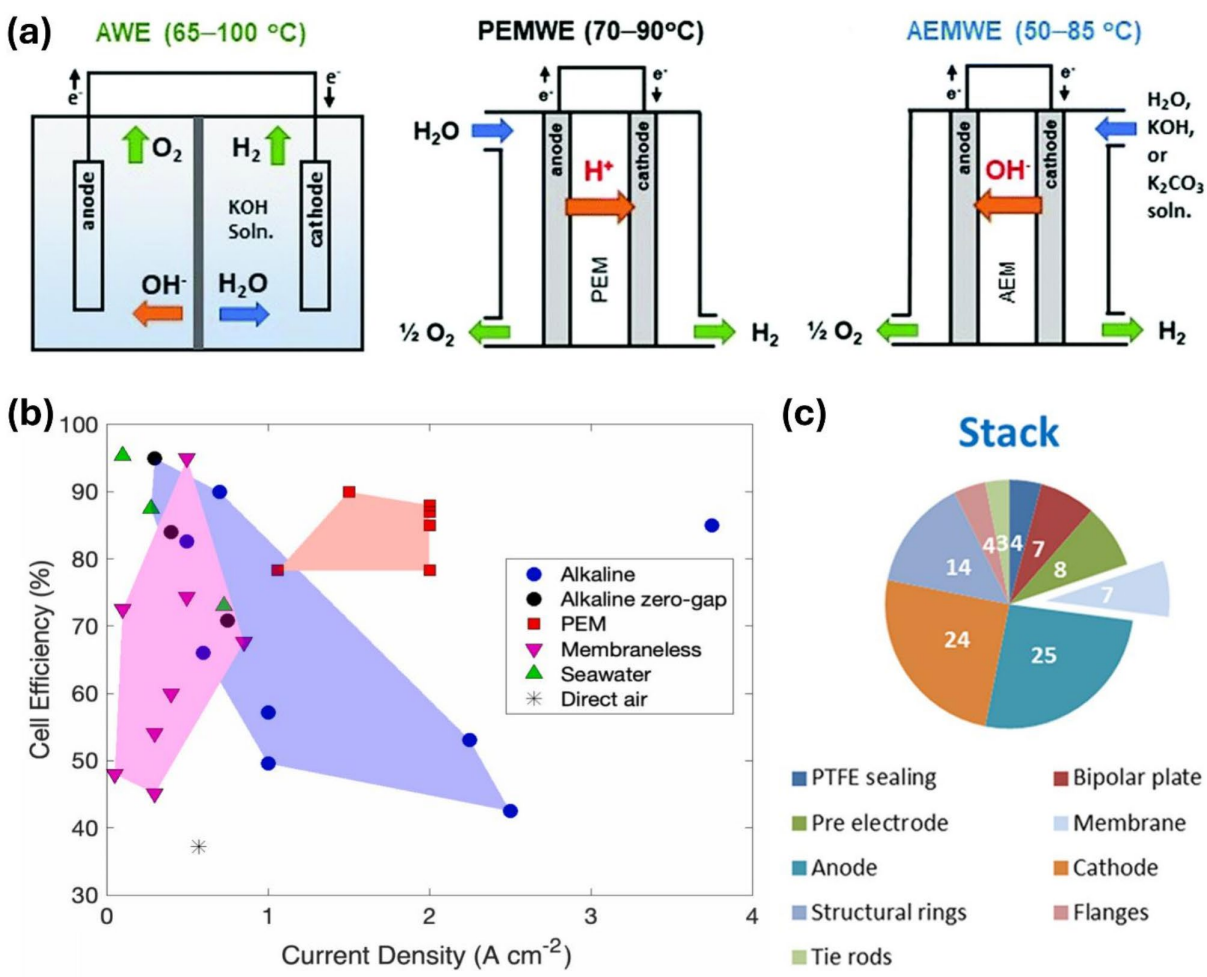
(**a**) Schematic configurations for different water electrolyzer single cells: AWE, PEMWE, and AEMWE. Images reproduced from Ref [1]. with permission from RSC; (**b**) Cell efficiency for various electrolyzers. Images reproduced from Ref [6]. with permission from Elsevier; (**c**) Stack cost breakdown (%) for an AWE system

Water electrolysis technologies have been utilized in commercial H_2_ production for several decades. A wide variety of low-temperature electrolyzer systems, including the proton exchange membrane water electrolyzer (PEMWE), alkaline water electrolyzer (AWE), and anion exchange membrane water electrolyzer (AEMWE), have been developed as renewable-energy-driven production methods for high-purity H_2_ generation without greenhouse gas emissions (Fig. 1a) [1]. Considerable development efforts over the past decade have focused on improving the production efficiency of H_2_, which is widely recognized as a green, renewable, and sustainable energy carrier that addresses environmental concerns and the substantial societal and industrial impacts of conventional fossil fuel usage. The basic reaction can be described by the following Eq. [2]:

1 $$\displaylines{1{H_2}O\, + \,(237.2\,kJ.mo{l^{ - 1}})\,Electricity\,\, \cr + \,(48.6\,kJ.mo{l^{ - 1}})\,Heat\, = \,{H_2} + 1/2{O_2} \cr}$$

The efficiency of H_2_ production via overall water splitting (OWS) is economically challenged due to significant energy consumption and a low H_2_ evolution rate. Solid oxide water electrolysis, conducted at high temperatures of 600–900 ^o^C using cost-effective metal (oxide) catalyst-based electrode materials, accelerates the kinetics for producing both H_2_ and O_2_ [3, 4]. However, it is hindered by serious gas crossover issues along with instability and degradation [5]. Meanwhile, PEMWE, AEMWE, and AWE, performed at lower temperatures of 30–80 ^o^C in either alkaline or acidic electrolyte solutions, face challenges in reaching commercial H_2_ production markets. The PEMWE offers energy efficiency up to 82%, rapid on/off operation, and a high pressure of 70 MPa for H_2_ production, alongside device lifetimes of approximately 50,000 h.

**Fig. 2:**
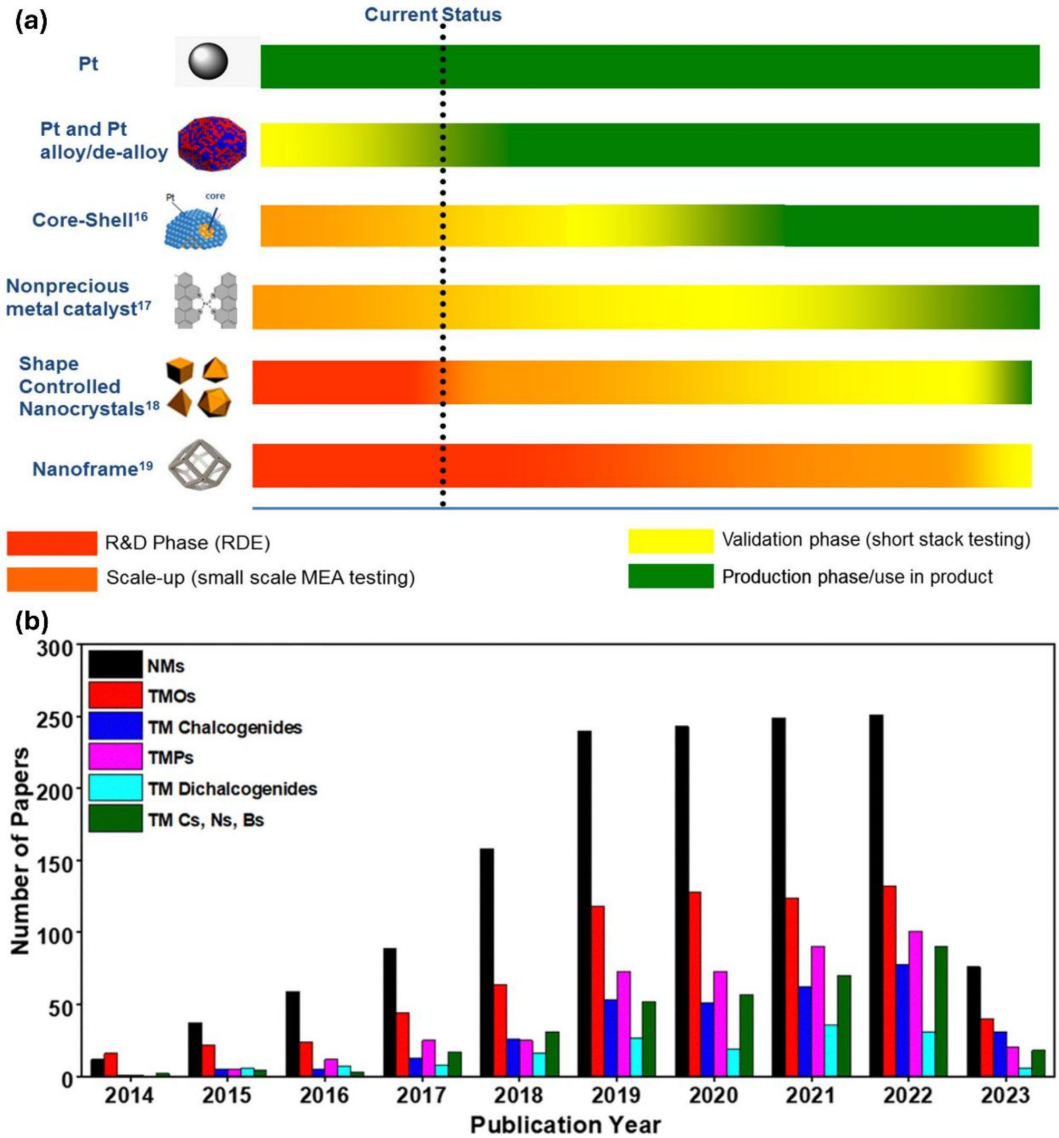
(**a**) Development timelines of electrocatalysts. Images reproduced from Ref [13]. with permission from ACS. (**b**) Recent publication progress of TM-based electrocatalysts for water splitting. Images reproduced from Ref [14]. with permission from the RSC

The AWE can achieve operating currents between 0.2 and 0.8 A cm^− 2^ and an energy conversion efficiency of 50–78%, with a lifetime of around 100,000 h, while most AEMWEs have a stated longevity of less than 3000 h [1, 5, 6]. The PEMWE has a lower H_2_ production capacity of less than 30 Nm^3^ h^-1^ with moderate efficiency, whereas AWE presents a better production capacity and efficiency (Fig. 1b). Thus far, the main challenges in these techniques include the high production costs and the need for efficiency improvements; consequently, significant research has been dedicated to enhancing the efficiency of components for OWS. Additionally, the cost breakdown of the system indicates that the stack accounts for half of the overall cost (Fig. 1c). Therefore, developing high-performance and cost-effective electrocatalysts to accelerate the sluggish hydrogen evolution reaction (HER) and oxygen evolution reaction (OER) in electrolyte solutions is considered a crucial approach to advancing OWS toward commercial viability in H_2_ markets. Extensive efforts from both academic labs and industrial companies have been directed towards developing active electrocatalysts for H_2_ production [7, 8]. In addition to expensive Pt-group metal (PGM)-based catalysts [9, 10], low-cost transition metal (TM) group-based candidates are seen as potential providers of promising activity and stability [11, 12]. As is well-known, the current limitation of electrocatalyst is still relied on heavily utilizing PGM and insufficient catalyst stability. To overcome the as-mentioned obstacles, various advances in structural engineering and surface composition tuning have emerged as highly promising solutions for creating advanced electrocatalysts with reduced or no PGM content (Fig. 2) [13, 14]. The interrelations between catalyst properties, each half-reaction, overall electrolyzer cell performance, and operating conditions have been evaluated for the successful potential of commercialization.

Therefore, an updated study on the current state of electrocatalyst engineering, highlighting both benefits and remaining challenges for OWS, should be considered. In this article, we will briefly discuss the current state and future tendencies of electrocatalysts for H_2_ production via electrochemical OWS. We systematically review developments relating to recent trends of catalysts from academic and industrial sectors and assess their application in industrial water electrolysis by leading companies. Finally, we identify several prospective engineering requirements to enhance the performance of future electrolyzer systems in response to the rapid growth of the global electrolyzer market for renewable H_2_ production.

## Current status and future of H2 energy carrier

**Fig. 3:**
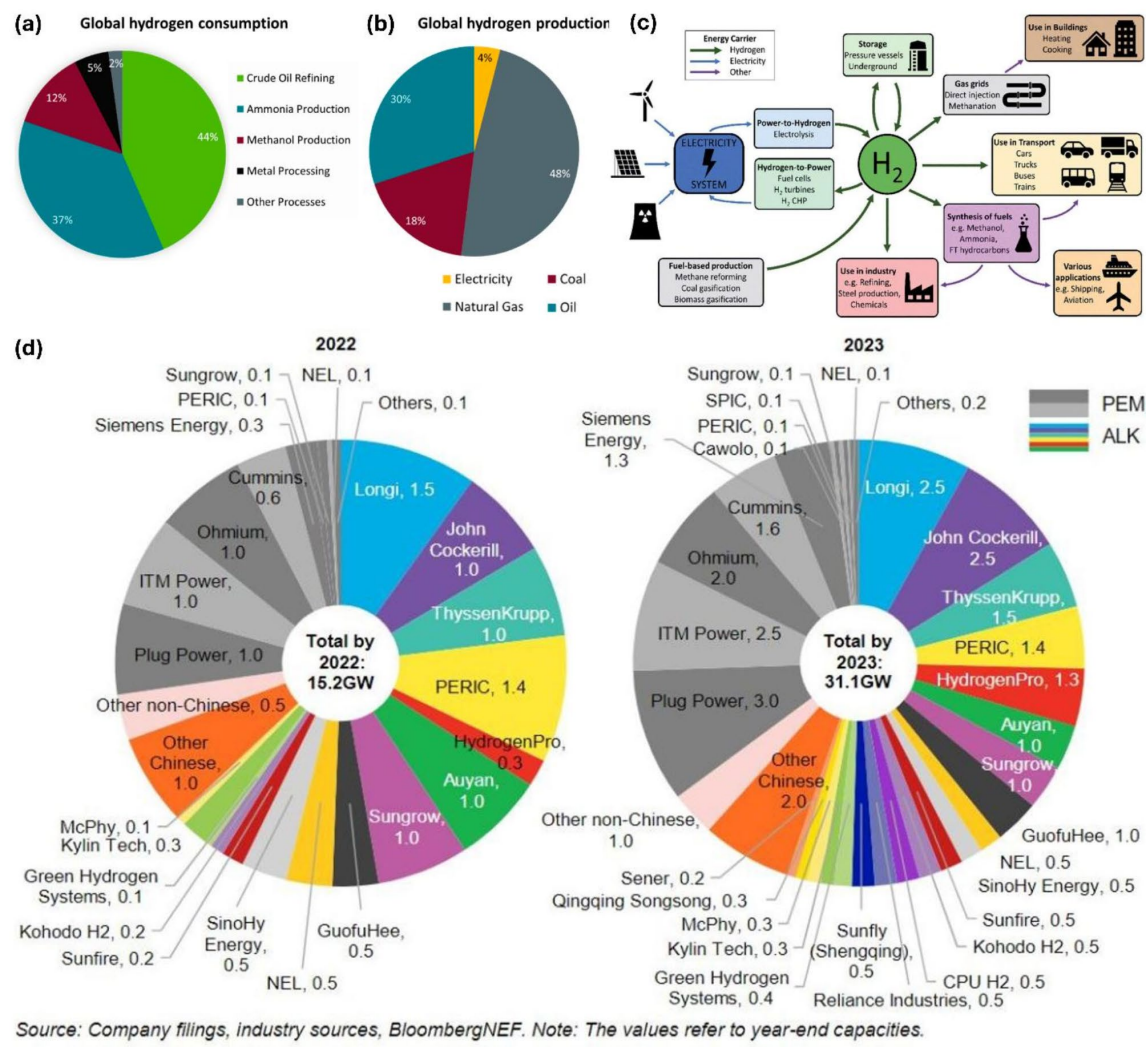
(**a**) Worldwide applications of hydrogen and (**b**) methods of production; (**c**) Future perspectives on hydrogen fuel and usage pathways; (**d**) Current electrolyzer manufacturing capacities [29]

Climate change is a principal driver for renewable energy development in the energy transition. The Intergovernmental Panel on Climate Change (IPCC) reported in 2018 that limiting global warming below 2 °C requires a decrease in CO_2_ emissions of around 25% by 2030 (from 2010 levels) and achieving global net zero by 2070 [15]. However, in contrast with this dream, CO_2_ emissions have recently continued to significantly grow and contributed around two-thirds of global greenhouse gas. An energy transition approach is now essential to decouple economic development from increased CO_2_ production. Consequently, the development of renewable energies for electricity generation in various industrial activities has gained considerable attention recently [16, 17]. The global renewable power capacity reached approximately 2537 GW in 2019 with the global renewable energy investment of around USD 303.5 billion in 2020 [18]. These energies accounted for 26.2% of global elctricity generation in 2018 and are expected to rise to 45% by 2040. Amog all renewable energy sources, H_2_ has emerged as a crucial part of the clean energy family, essential for ensuring a sustainable future [19] due to its renewability, high energy storage capacity, and zero emission when combusted [20]. In recent years, two significant advances have greatly promoted H_2_ development: the cost of H_2_ produced by renewables has decreased and will continue to decline, while concerns related to greenhouse gas emissions have urgently increased; as a result, many countries are actively transitioning towards non-carbon economies, especially in energy source and demand. The application of H_2_ has expanded to various sectors, including automotive, energy-intensive industries, heating, maritime transport, aviation, and trucking (Fig. 3a) [21, 22]. Over the next few decades, H_2_ is expected to comprise about 18% of total energy use by 2050. Although H_2_ plays a critical role in the global energy transition, sourcing remains a challenge. Currently, natural gas and coal are the primary sources for generating about 96% of all H_2_ energy carriers [23, 24]. There is negligible H_2_ production from renewable pathways [24, 25], although producing green H_2_ through this method is nearly a zero-carbon production approach. In this context, only about 4% of H_2_ is produced through electrolysis, resulting in H_2_ and O_2_ gases (Fig. 3b) [26]. However, due to the significant role of clean H_2_ in meeting future global energy demands, an unprecedented collaboration of political and business strategies is essential to enhance production technologies, lower costs for broader implementation, and reduce greenhouse gas emissions (Fig. 3c) [26]. Efforts are being intensified in multiple countries to boost the use of green H_2_ through the adoption of green energy carriers produced by OWS, splitting into H_2_ and O_2_ using electrolyzers powered by electricity (Fig. 3d) [7, 27, 28]. There has been widespread construction of larger, more efficient electrolysis systems that are better suited for power systems, as reported in multiple studies. For instance, since 2017, Germany has commissioned a 6-megawatt (MW) electrolyzer in Mainz, complemented by a 10 MW PEM electrolyzer near Cologne in 2020. In the Netherlands, a 2 GW electrolyzer system has been constructed (DI, 2019). In early 2020, plans were made to construct a 20 MW electrolyzer with potential expansion to 60 MW by year’s end. In Austria, Siemens has supplied a 6 MW PEM electrolyzer. Meanwhile, Toshiba Co. has set up a 10 MW electrolyzer in Japan, which is expected to produce 900 tons of H_2_ annually for transportation applications.

Japan’s strategy also involves a power-to-gas system incorporating a 1.5 MW PEM electrolysis facility integrated with a 21 MW solar photovoltaic system. In Australia, a 50 MW electrolyzer has been established as part of a new H_2_ center near Crystal Brook. These pioneering commercial projects demonstrate a strong global interest in producing H_2_ through water electrolysis, which is considered the optimal renewable H_2_ source for many emerging applications. While electrolyzers are scaling up from MW to gigawatt (GW) levels, technological advancement remains gradual, primarily due to high investment costs of approximately 840 USD/kW for AWE, 1200 USD/kW for PEMWE, or 444 USD/kW for AEMWE [30–32]. The cost to produce 1 kg green hydrogen in western Europe is currently in the range of roughly 3.2–8.2 USD, still much higher than 1.5–1.8 USD/kg of grey H_2_ [30]. Therefore, to be competitive, renewable H_2_ needs to be produced at less than 2.5 USD/kg and it is estimated a low-cost electrolyzer costing < 100 USD/kW for 1 MW system or < 200 USD/kW for 20 MW will be achieved by 2050 [32]. Renewable H_2_ via OWS will become the most cost-effective clean H_2_ source if its current limitations are swiftly addressed. The challenges in electrode materials, membranes, operating conditions, and stack assembly approaches are crucial, with electrolyzers still in the burgeoning phase of R&D. Among these, research on optimizing electrocatalysts for both cathodic and anodic electrodes is vital for enhancing the efficiency of commercial electrolyzers, ensuring they are reliable, efficient, robust, and cost-effective for H_2_ production.

## Current engineering state of electrocatalysts for OWS

**Fig. 4:**
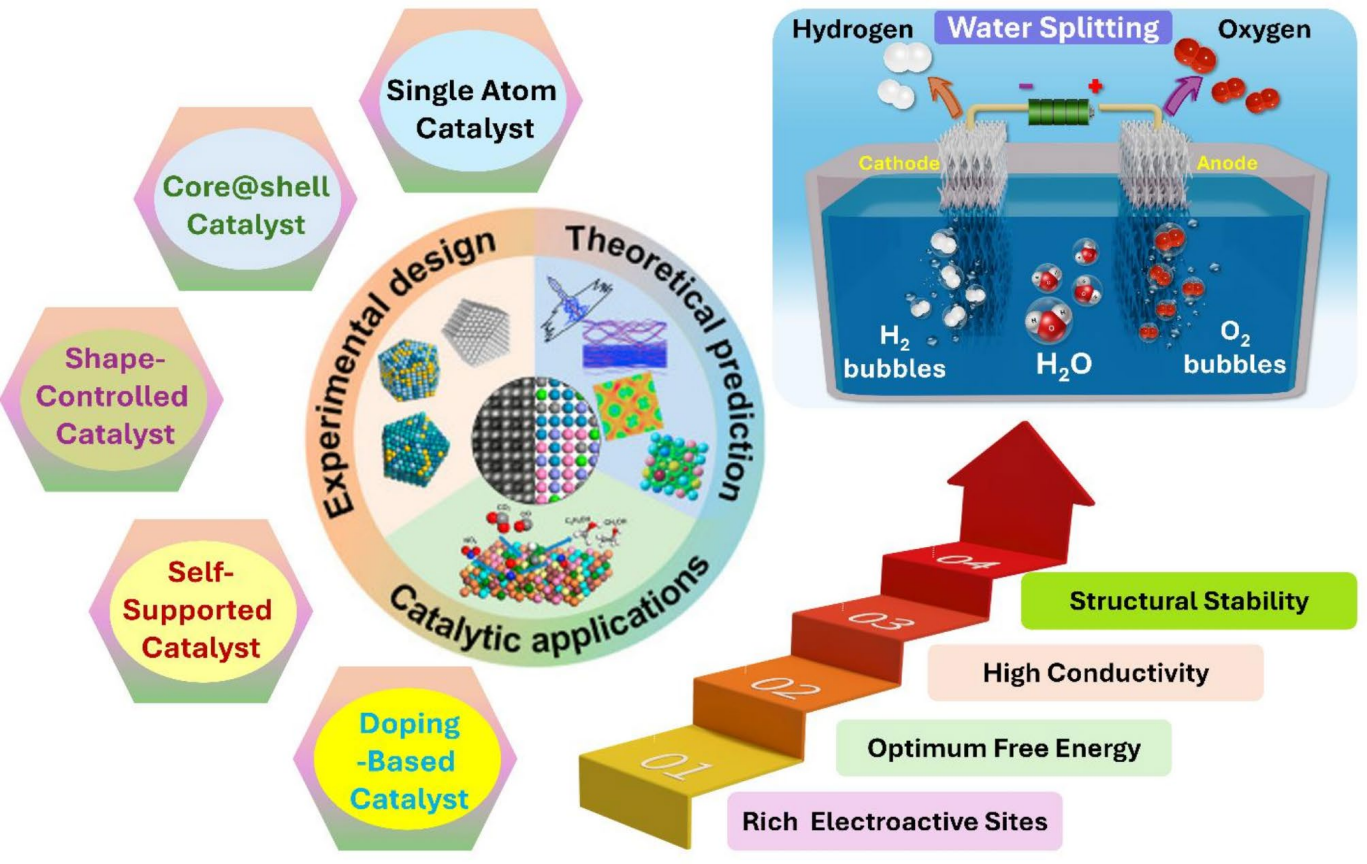
Different catalyst engineering approaches for electrocatalyst development. Images reproduced from Ref [38]. with permission from the Elsevier

Electrocatalysts are essential for the efficiency of commercial water splitting systems as they reduce the kinetic overpotential (η) of the HER and OER. The slow and complex charge transfer rate from multi-step electrochemical reactions originating between the reactant and the electrode surface necessitates an η value to overcome the activation barrier. In electrolyzers, electrocatalysts are directly deposited onto both anode and cathode electrodes for water oxidation and reduction, respectively [33–35]. An effective catalyst provides excellent catalytic activities characterized by high current density and low η value and enhances the stability/durability of the electrolyzer system, significantly reducing the total cost of H_2_ production and utilization. Researchers worldwide are vigorously developing water-splitting catalysts using varied principles and exploring distinct structure–function relationships to establish guidelines for optimizing the reaction’s energy efficiency (Fig. 4) [36–38]. Several main engineering approaches for the development of effective catalysts towards OWS is specifically discussed in detail in the next sections.

### Single-atom electrocatalysts (SACs)

**Fig. 5:**
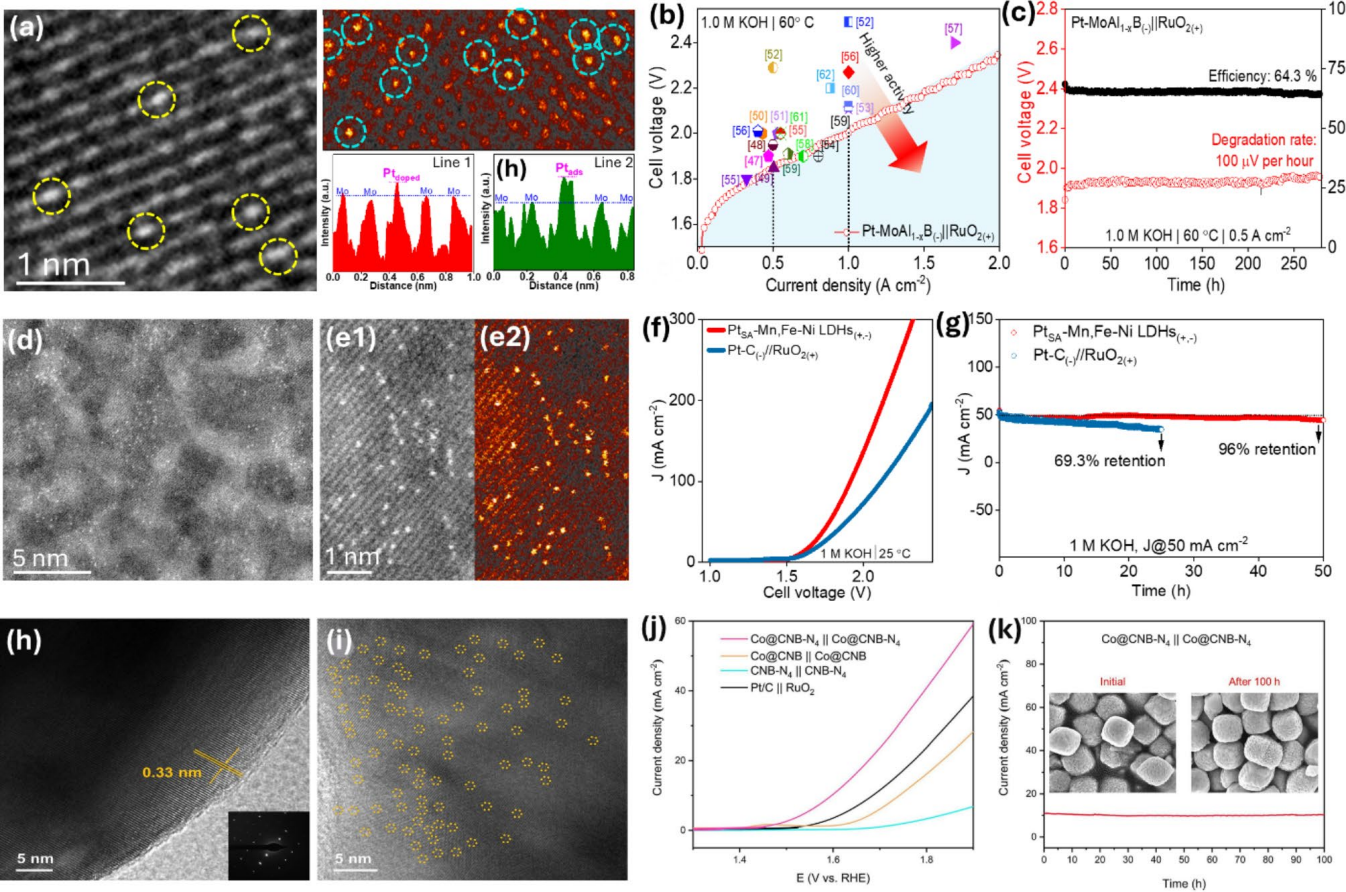
(**a**) Structure of the Pt-MoAl_1 − x_B catalyst and (**b**-**c**) OWS performance of the Pt-MoAl_1 − x_B//RuO_2_ couple. Images reproduced from Ref [42]. with permission from the RSC; (**d**-**e**) Structure of the Pt_SA_–Mn, Fe–Ni LDHs catalyst and (**f**-**g**) OWS performance of the Pt_SA_–Mn, Fe–Ni LDHs_(+,-)_ couple. Images reproduced from Ref [43]. with permission from the ACS; (**h**-**i**) Structure of the Co@CNB-N_4_ catalyst and (**j**-**k**) OWS performance of the Co@CNB-N_4(+,-)_ couple. Images reproduced from Ref [44]. with permission from Elsevier

Reducing the size of nano-catalysts to the atomic level has proven to be highly effective in achieving advanced HER and OER performances that are comparable to or surpass benchmark catalysts. SACs consist of isolated metallic atoms on suitable support materials, offering unique properties such as atomic dispersion of metal centers that maximize active sites and mass activity, coordinatively unsaturated metal active sites, quantum size effects, and strong interactions with the support matrix [39]. These distinctive properties of SACs can simultaneously enhance the catalytic activity, stability, and selectivity of the materials for various electrochemical reactions. For water electrolysis, constructing SACs on 0D, 1D, 2D, and 3D support materials, including nanocarbon and metal-based materials, has shown great promise for practical water splitting applications. However, to avoid the aggregation effect and maintain the mono dispersion of atomic structures on supports, the concentration of metal atoms is usually kept below 1.5 wt%, which limits the mass/volume activity of the developed catalysts [40, 41]. In order to provide comprehensive information about the structure and properties of SACs, such as the coordination environment, verification of single atoms, and chemical/electronic states of metal atoms, specialized advanced characterizations like scanning transmission electron microscopy (STEM), X-ray absorption (XAS) which includes X-ray absorption near-edge structure (XANES) and extended X-ray absorption fine structure (EXAFS) techniques, along with theoretical density functional theory (DFT), have been employed to delve into the nature of the catalysts and allow for the precise design of potential catalysts with tailored activities. Initial efforts initiated a focused exploration of SACs with enhanced electrocatalytic activities and stability towards HER and OER for electrocatalytic water splitting application. Park et al. utilized a quantity of isolated Pt single atoms immobilized on layered ternary transition metal boride nanosheets–MoAl_1 − *x*_B, which displayed extensive exposure of basal planes and Mo-vacancy defect sites [42]. The authors confirmed the presence of Pt SAs on the surface of MoAl_1 − *x*_B *via* adsorption and doping behaviors created by electronic coupling between Pt and B/Mo atoms (Fig. 5a). The charge relocation and electronic reconfiguration in doped and adsorbed Pt SAs (Pt_doped_ and Pt_ads_.) enhance adsorption and activation towards H_2_O. Consequently, the Pt-MoAl_1 − x_B catalyst significantly improves HER activity in both alkaline and acidic media, achieving low overpotential (η) values of 32 and 18 mV to reach 10 mA cm^− 2^ respectively (Fig. 5b). An electrolyzer cell using a Pt-MoAl_1 − *x*_B cathode can sustain an industrial-level current density of 0.5 A cm^− 2^ and 1.0 A cm^− 2^ at cell voltages of 1.85 and 2.0 V, respectively, in 1.0 M KOH at 60 °C, demonstrating good efficiency and durability (Fig. 5c). The exceptional catalytic HER performance of the Pt-MoAl_1 − *x*_B suggests it is a highly effective HER catalyst for practical water electrolysis applications. A study reported by Tran et al. introduced a controllable engineering technique to alter the Ni(OH)_2_ structure using Pt_doped_ and Pt_ads_. enhanced by the low electronegativities of Mn and Fe moieties (Pt_SA_–Mn, Fe–Ni LDHs) [43]. The author explored the uniform dispersion of Pt, Mn, and Fe on the Ni LDH surface results in the charge donation from transition metal moieties into Pt (Fig. 5d-e). This adjusts the Pt *d*-band center to effectively facilitate H_2_ release, while altered local chemical environments at TM sites primarily function in dissociating H_2_O into O_2_. Thus, the Pt_SA_–Mn, Fe–Ni LDH material only requires a small η of 42 and 288 mV to achieve 10 mA·cm^–2^ for HER and OER, respectively, outperforming commercial Pt-C//RuO_2_ and recently documented LDH-based electrocatalysts (Fig. 5f-g). In another study, Li et al. found a balance between enhancing atom loading and preventing the agglomeration of metal SAs [44]. They prepared Co SAs embedded in *N*-doped carbon nano boxes (Co@CNB-N_4_), where solid bonds between the Co SAs and the carbon matrix ensure uniform dispersion of atomic Co with a high mass loading of ∼10.2 wt% (Fig. 5h-i).

The presence of Co SAs in Co@CNB-N_4_ results in the η10 of 45 mV for HER and 250 mV for OER, culminating in a cell voltage of 1.59 V to generate a current density of 10 mA cm^− 2^. This performance is lower compared to that of RuO_2_//Pt/C and includes long-term stability over 100 h without significant degradation of performance (Fig. 5j-k). Chen et al. suggest that the introduction of various non-metallic atoms into the supporting matrix can effectively alter the electronic environment around the central metal SAs, thereby enhancing the electrocatalytic activity [45]. Moreover, compared to mono SACs, dual SACs demonstrate superior catalytic activities due to a more complex atomic coordination environment and synergistic catalysis effects. These enhancements effectively lower the energy barrier and improve the reaction kinetics, as demonstrated by Fang et al. with Fe, Mo dual SAs-modified CoNi-based nanosheets [46], Ge et al. with Ru, Ni-co-modified MoS_2_ [47], and Wang et al. with Fe, Pt SAs-supported N-C [48]. Various studies have reported the fabrication of isolated metal SAs anchored on substrates with diverse coordination environments, exhibiting superior catalytic activity, selectivity, and maximum metal utilization for HER and OER [49–55]. Despite the intriguing achievements of SACs, significant challenges still remain during their development, including: (i) Insufficient metal SAs loading with agglomeration makes low efficiency of atom utilization; (ii) Limited understanding of the reliable bonding and interactions between SAs and matrix, which is crucial for optimal electron distribution and to enhance both the electrocatalytic performance and durability of the catalysts. Therefore, future research priorities should focus on developing novel engineering approaches to balance between increasing the SAs amount while maintaining high efficiency. Additionally, employing theoretical techniques and in-situ characterizations is crucial to explore the specific physicochemical interactions between SAs and matrix and their effect on catalytic performance.

### Core@shell electrocatalysts

**Fig. 6:**
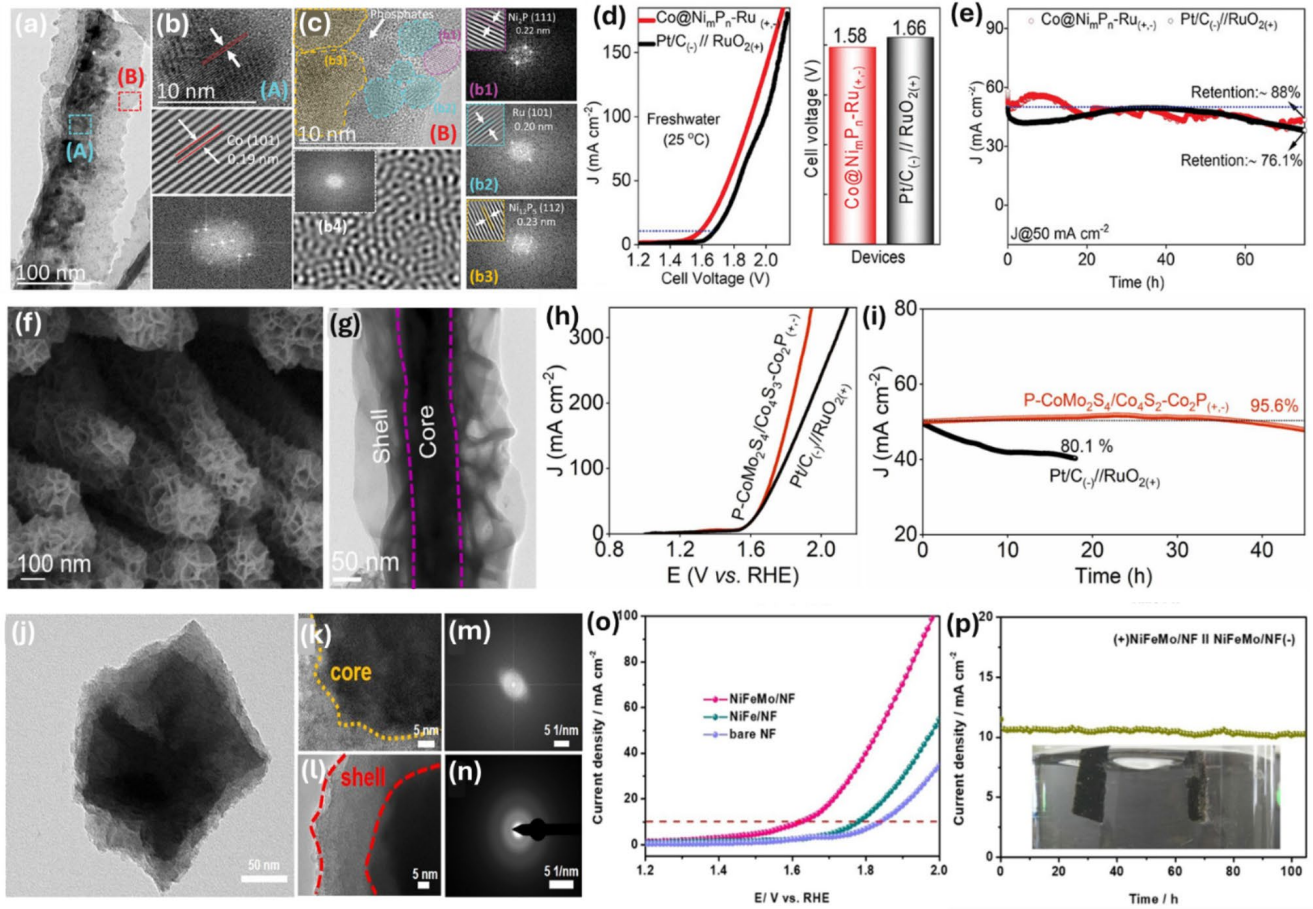
(**a**-**c**) Structure of the Co@Ni_m_P_n_–Ru catalyst and (**d**-**e**) OWS performance of the Co@Ni_m_P_n_–Ru_(+,-)_ couple. Images reproduced from Ref [61]. with permission from Wiley; (**f**-**g**) Structure of P − CoMo_2_S_4_/Co_4_S_3_–Co_2_P catalyst and (**h**-**i**) OWS performance of the P − CoMo_2_S_4_/Co_4_S_3_–Co_2_P_(+,-)_ couple. Images reproduced from Ref [62]. with permission from Elsevier; (**j**-**n**) Structure of NiFeMo films and (**0**-**p**) OWS performance of the NiFeMo_(+,-)_ couple. Images reproduced from Ref [63]. with permission from Elsevier

To reduce the use of precious metals and develop unique physicochemical properties of materials for catalytic reactions, surface and interface engineering is crucial. The formation of a core@shell nanostructure by combining a core material with a shell material is promising for enhancing the efficiency and long-term stability of catalysts for water splitting. Additionally, such core@shell nanostructures can lower catalyst costs and facilitate strong interactions between components, resulting in a hybrid with greater activity and stability compared to simple noble/nonnoble metal nanostructures [56]. This improvement is because the design of core@shell nanostructures is a key strategy to expose active sites, increase site multiplicity, and significantly alter electronic/chemical structures, thereby enhancing performance and creating synergistic improvements in electrochemical working mechanisms. The development of metal-metal core-shell and metal-2D carbon core@shell materials has been extensively studied for water electrolysis. For the synthesis of core-shell nanostructures, the process primarily involves bottom-up methods, such as chemical reduction reactions, solvo/hydrothermal reactions, chemical vapor deposition (CVD), and galvanic replacement reactions. By controlling reaction conditions, including initial precursor content, reaction order, reaction temperature, and surfactant types/concentration, it is possible to tailor size, shape, morphology, structure, and textural properties easily and effectively. Various 0D, 1D, and 2D metal-metal core-shell structures have been synthesized, showing promising catalytic properties for water electrolysis. Recently, Guo and colleagues prepared a Co_3_S_4_@MoS_2_ core@shell hollow cubic heterostructure through a two-step hydrothermal reaction [57]. The structural advantages of hollow and core-shell configurations, composed of dense internal Co_3_S_4_ nano boxes and external MoS_2_ nanosheets (NSs), provided strong interfacial coupling between components, leading to synergistic effects for active and stable HER and OER performance with low η values of 280 and 136 mV for OER and HER at 10 mA cm^− 2^ in 1.0 M KOH solution. Consequently, a low cell voltage of 1.58 V was achieved when the catalyst was used as the anodic and cathodic electrodes for water electrolysis. To reduce the overpotentials of catalytic HER and OER, Zhu et al. introduced an efficient synthesis approach using electrospun Co–carbon nanofibers (NFs) as templates for a hydrothermal step followed by a sulfurization procedure to synthesize CoS_2_–C@MoS_2_ 1D core@shell NFs-based catalysts [58]. The CoS_2_-C@MoS_2_ 1D core@shell NFs displayed a strong interface between MoS_2_ NSs and CoS_2_-C NFs, leading to heightened charge transfer and thus superior reaction kinetics, requiring a η of approximately 173 mV at 10 mA cm^–2^ and demonstrating stability over 1000 cycles. Meanwhile, Xie and colleagues reported the in-situ electrochemical design of an ultrathin Ni–Bi nanolayer on metallic Ni_3_N NSs, creating a durable 2D core@shell structured nanoarrays supported by a Ti mesh (Ni_3_N@Ni–Bi NS/Ti) [59]. The NS arrays, approximately 2.8 μm thick, required a cell voltage of 1.95 V to deliver 10 mA cm^− 2^ in 0.5 M K–Bi solution at 25 °C. Yu et al. reported on a novel hierarchical 1D Cu@CoFe LDH core@shell catalyst for efficient overall water splitting in an alkaline medium [60]. Malhotra et al. designed a triphasic heterostructure comprising Ni_2_P–Ni_12_P_5_–Ru with amorphous interface engineering, robustly coating over a cobalt nano-surface (Co@Ni_m_P_n_–Ru) to construct a hierarchical 3D interconnected core@shell architecture through a combination of hydrothermal reaction, electrodeposition, and thermal treatment processes [61]. The formation of the triphasic Ni_2_P–Ni_12_P_5_–Ru structure established numerous heterojunctions with lattice mismatch and defects, thereby exposing more electroactive sites with well-tuned electronic properties (Fig. 6a-c). The optimum free energy achieved by the Co@Ni_m_P_n_–Ru structure elevated the catalytic prowess, with the required η of only 30 mV for HER at 10 mA cm^− 2^ and 320 mV for OER at 50 mA cm^− 2^ in freshwater, thus allowing the corresponding two-electrode electrolyzer cell to achieve a low cell voltage of just 1.43 V in alkaline freshwater, reaching 10 mA cm^− 2^ at 80 °C with excellent activity retention over 76 h (Fig. 6d-e). In another study, Dong et al. prepared a core@shell structure based on P-doped CoMo_2_S_4_ coupled Co_4_S_3_/Co_2_P (P − CoMo_2_S_4_/Co_4_S_3_–Co_2_P) to form a three-phase interface catalyst [62].

The material was constructed through several applications of hydrothermal reaction and thermal treatments, resulting in a nanolayer of CoMo_2_S_4_ sheets uniformly coating over the 1D Co_2_P–Co_4_S_3_ surface (Fig. 6f-g). The catalyst demonstrates exceptional physicochemical properties, including a large specific surface area, numerous active centers, and excellent conductivity, thus serving as a high-performance, binder-free, self-supporting electrode that enhances both HER and OER in an alkaline medium. It achieves a current density of 10 mA cm^− 2^ at an overpotential η of just 54 mV for HER and 296 mV for OER. Consequently, a two-electrode electrolyzer based on P − CoMo_2_S_4_/Co_4_S_3_–Co_2_P_(+,−)_ could sustain a minimal cell voltage of 1.55 V at 10 mA cm^− 2^ with 95.6% performance retention after 50 h of water splitting, producing green hydrogen gas (Fig. 6h-i). Recently, Wang et al. introduced a bifunctional electrocatalyst with a core@shell structure, consisting of amorphous NiFeMo films in-situ grown on a nickel foam (NF) substrate via a straightforward hydrothermal process and ultrasonic treatment. Supported on the NF surface, the nano catalyst exhibits a core@shell structure without distinct lattice fringes in either the core or shell, due to their amorphous nature (Fig. 6j-n) [63]. This catalyst achieved an overpotential η of 215 mV for OER and 180 mV for HER at 10 mA cm^− 2^. As a result, coupled electrodes derived from NiFeMo/NF(+,-) require only a cell voltage of 1.62 V to achieve 10 mA cm^− 2^ and exhibit excellent stability exceeding 100 h during overall water-splitting in an alkaline medium (Fig. 6o-p). In another research, Han et al. also prepared well-distributed core@shell nanoparticles of Co_9_S_8_@MoS_2_ on porous carbon nanofibers [64]. The Co_9_S_8_@MoS_2_/CNFs exhibited highly efficient HER and OER performance, demonstrating a low cell voltage of 1.41 V at 10 mAcm^-2^ towards complete water splitting and remarkable long-term stability. Various studies have highlighted the intricate design of core@shell heterostructures, along with elemental doping and interfacial engineering, to enhance the practical application of electrocatalytic water splitting [65–68]. These achievements in developing core@shell catalysts underscore their benefits in boosting catalytic performance for water splitting, characterized by improved activity and stability through several mechanisms. From the aforementioned advancements in the development of core@shell catalysts, it is apparent that their benefits, which lead to improved catalytic performance in water splitting, can be attributed to several factors: (i) The robust shell not only effectively protects active cores during catalyst fabrication and electrochemical reactions but also exposes numerous active sites, enhancing activity and ensuring long-term stability [69, 70]; (ii) The unique interactions between the core and shell parts induce lattice strain in the shell, interfacial electronic redistribution, and modification of d-band centers, achieving optimal electronic/chemical configurations for active sites, thus optimizing the free energy and enhancing intrinsic activity [71–74]; (iii) The formation of diverse active sites on core@shell structures may provide a synergistic effect that enhances activity during the multiple-step processes of HER and OER [75, 76]. Although various feasible core@shell nanostructure-based electrocatalysts have been explored to improve activity and stability for water splitting, electrocatalytic performance heavily relies on the synthetic approach, posing significant challenges in regulating core@shell interfaces, which necessitates innovative and universal catalyst preparation strategies. Moreover, deeper understanding of the connections between physicochemical properties and the catalytic mechanism of core@shell nanostructures is crucial for further enhancing the efficiency of electrocatalysts to meet practical water splitting demands.

### Shape-controlled electrocatalysts

**Fig. 7:**
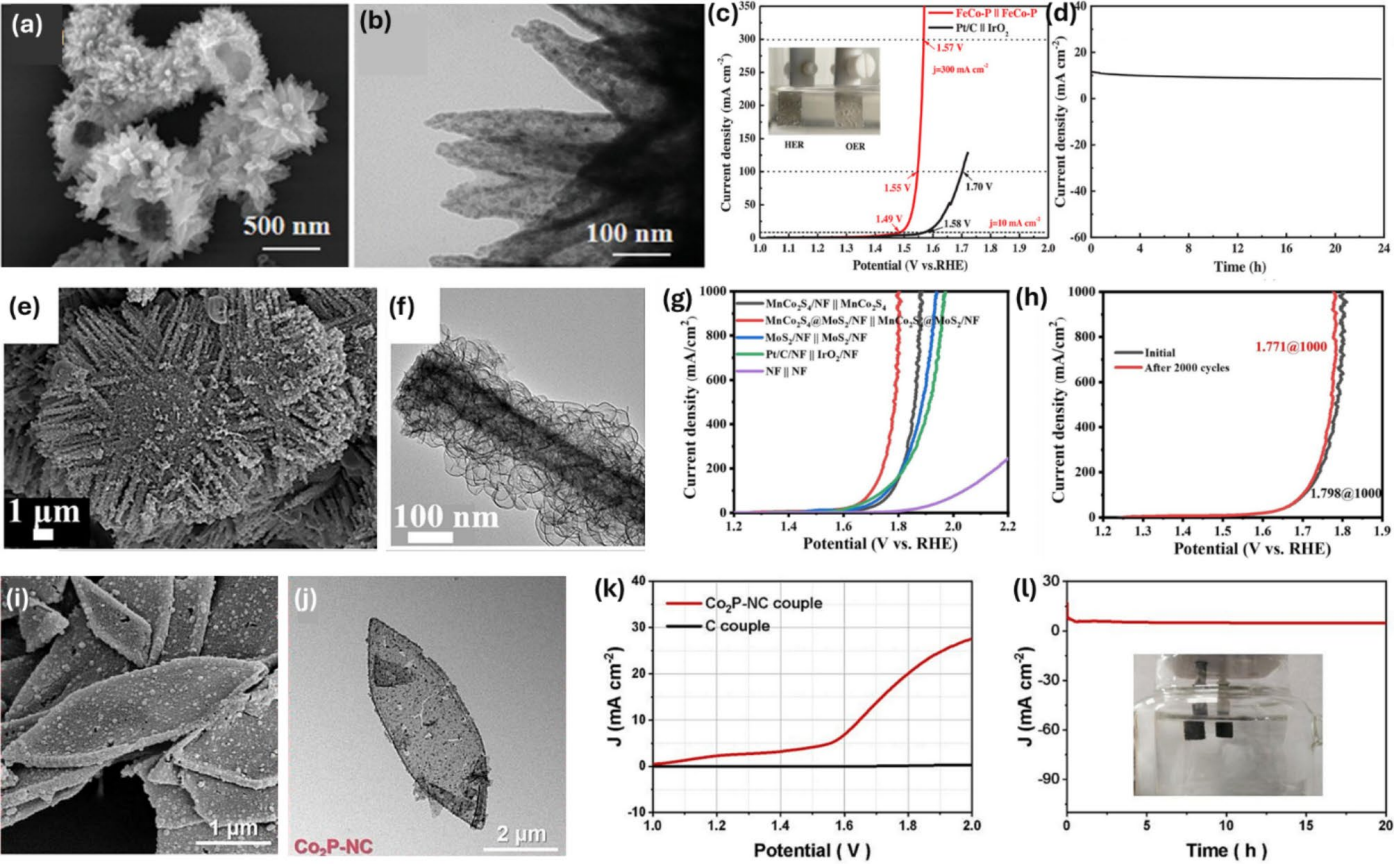
(**a**-**b**) Structure of FeCo-P hollow spherical heterostructures and (**c**-**d**) OWS performance of the FeCo-P_(+,-)_ couple. Images reproduced from Ref [82]. with permission from Wiley; (**e**-**f**) Structure of the MnCo_2_S_4_@MoS_2_/NF catalyst and (**g**-**h**) OWS performance of the MnCo_2_S_4_@MoS_2_/NF_(+,-)_ couple. Images reproduced from Ref [84]. with permission from Elsevier; (**j**-**n**) Structure of leaf-like Co_2_P-NC and (**o**-**p**) OWS performance of the Co_2_P-NC_(+,-)_ couple. Images reproduced from Ref [85]. with permission from Elsevier

Although researchers have recently focused on studying and optimizing various electrocatalyst materials to enhance their efficiency and cost-effectiveness for water splitting, challenges remain due to the relatively low catalytic activity caused by kinetically sluggish HER and OER steps. Tuning the morphology and structure of these materials offers several advantages. First, it can increase the surface area-to-volume ratio, enhancing the number of available active sites for catalysis [77]. Additionally, forming 1D, 2D, or 3D morphologies can enhance charge transport pathways and provide multiple channels for effective diffusion of reactants/products, thereby reducing mass transfer limitations and improving reaction rates [78, 79]. Moreover, controlled morphologies contribute to high structural stability, preventing the aggregation or leaching of active catalyst species during operation, thus ensuring long-term durability [80, 81]. Hollow nanostructures have garnered significant interest in electrochemical water splitting due to their unique structural properties, which include a high surface-to-volume ratio, rapid charge transfer, and favorable mass diffusion. In this context, Jiang et al. successfully synthesized a hollow spherical heterostructure of FeCo-P through hydrothermal and phosphidization processes [82]. This hollow spherical FeCo-P catalyst, composed of nanosheets with a diameter of approximately 1 μm, features a heterogeneous interface between Co_2_P and FeP phases (Fig. 7a-b). The resulting hollow structure not only facilitates mass and electron transfer but also increases accessible active sites and the contact area between the catalyst and the electrolyte, leading to enhanced catalytic properties. Thus, FeCo-P exhibits high HER and OER performance with η values of 131 and 240 mV, respectively, to reach 10 mA cm^− 2^ in 1 M KOH. As FeCo-P serves as both the cathode and anode in OWS, only a low voltage of 1.49 V is required to achieve 10 mA cm^− 2^ (Fig. 7c-d). In another study, Su et al. synthesized CoO-Mo_2_N hollow heterojunctions, which present as uniform rhombic dodecahedra with a slightly rough surface [83]. Driven by the synergistic effects of hollow structures and heterojunctions, CoO-Mo_2_N demonstrates excellent HER activity, achieving an η of 65 mV at 10 mA cm^− 2^ in 1 M KOH. Ma et al. developed unique MnCo_2_S_4_ hexagonal stars coated with MoS_2_ NSs grown on an NF substrate (MnCo_2_S_4_@MoS_2_/NF) (Fig. 7e-f) [84]. This heterostructure, with its optimized composition and local electronic structure, offers rich active sites and heterointerfaces, thus effectively boosting electron transfer and promoting reaction kinetics.

The successful construction of this heterostructure as a bifunctional catalyst achieves required η of 208 and 332 mV in 6.0 M KOH to drive 1000 mA cm^− 2^ for HER and OER, respectively. Furthermore, the MnCo_2_S_4_@MoS_2_/NF-based electrolyzer cell for OWS requires only 1.795 V to deliver 1 A cm^− 2^ with reliable stability (Fig. 7g-h). Zhang et al. also discovered that modifying the Mo/Co ratio could regulate the structure of the resulting material into solid/hollow cubes through a simple synthesis method [86]. Consequently, an adjusted electronic structure was achieved by the upward shift of the d-band for Mo and downward shift for Co, thereby enhancing both HER and OER processes with a minimal η of 223 mV and 52 mV at 10 mA cm^− 2^, respectively. The electrolytic cell based on this catalyst requires only 1.53 V to reach 10 mA cm^− 2^. Recently, Xue et al. introduced a low-cost and robust bifunctional TM phosphide-based material, hollow leaf-like Co_2_P/N-doped porous carbon (Co_2_P-NC), through a chemical reaction followed by a pyrolysis process [85]. This controlled strategy in structures and compositions leads to numerous Co_2_P NPs anchored within a framework of hollow N-rich porous carbon nano-leaves, providing abundant highly accessible active sites due to effective aggregation inhibition (Fig. 7i-j). Benefiting from this unique 2D hollow porous architecture and abundant Co_2_P active species, the as-prepared Co_2_P-NC shows excellent activity and stability towards HER and OER with η of 31 and 349 mV, respectively, resulting in OWS performance with an overall voltage of 1.648 V at 10 mA cm^− 2^ and stability lasting 20 h in alkaline conditions (Fig. 7k-l). Meanwhile, Wang et al. successfully prepared 3D porous spherical nanoflowers of Co_6_Ni_4_P/NF through a one-step pulse electrodeposition process [87]. The morphology of the synthesized material combines the advantages of 3D spherical and 2D sheet structures to create a large specific surface area and a high-density of active sites, thereby enabling the OWS to achieve 50 mA cm^− 2^ at a cell voltage of only 1.56 V. The regulation of morphology and composition to create a variety of structures, such as cubes, hollow particles [88–90], nanotubes [91], hollow nanorods [92], nano boxes [93–95], hollow spheres [96], nano frames [97], nanocages [98, 99], and 3D flower-like nanostructures [100–103] for enhancing catalytic activity towards OWS, was also documented in recent studies. So far, significant progresses in shape-controlled electrocatalysts have been developed, challenges relating to synthetic approaches for precise control of size and shape and ensuring the stability of shape-controlled electrocatalysts to prevent collapse or distortion during reactions are still the urgent issues. The successful development of new methods is necessary in understanding and controlling the surface structures of electrocatalysts, facilitating the rational design of advanced electrocatalysts. Moreover, the formation of hybrid architectures based on the combination of 1D, 2D, and 3D shapes is also crucial to maximize the active centers and structural stability.

### Self-supported electrocatalysts

**Fig. 8:**
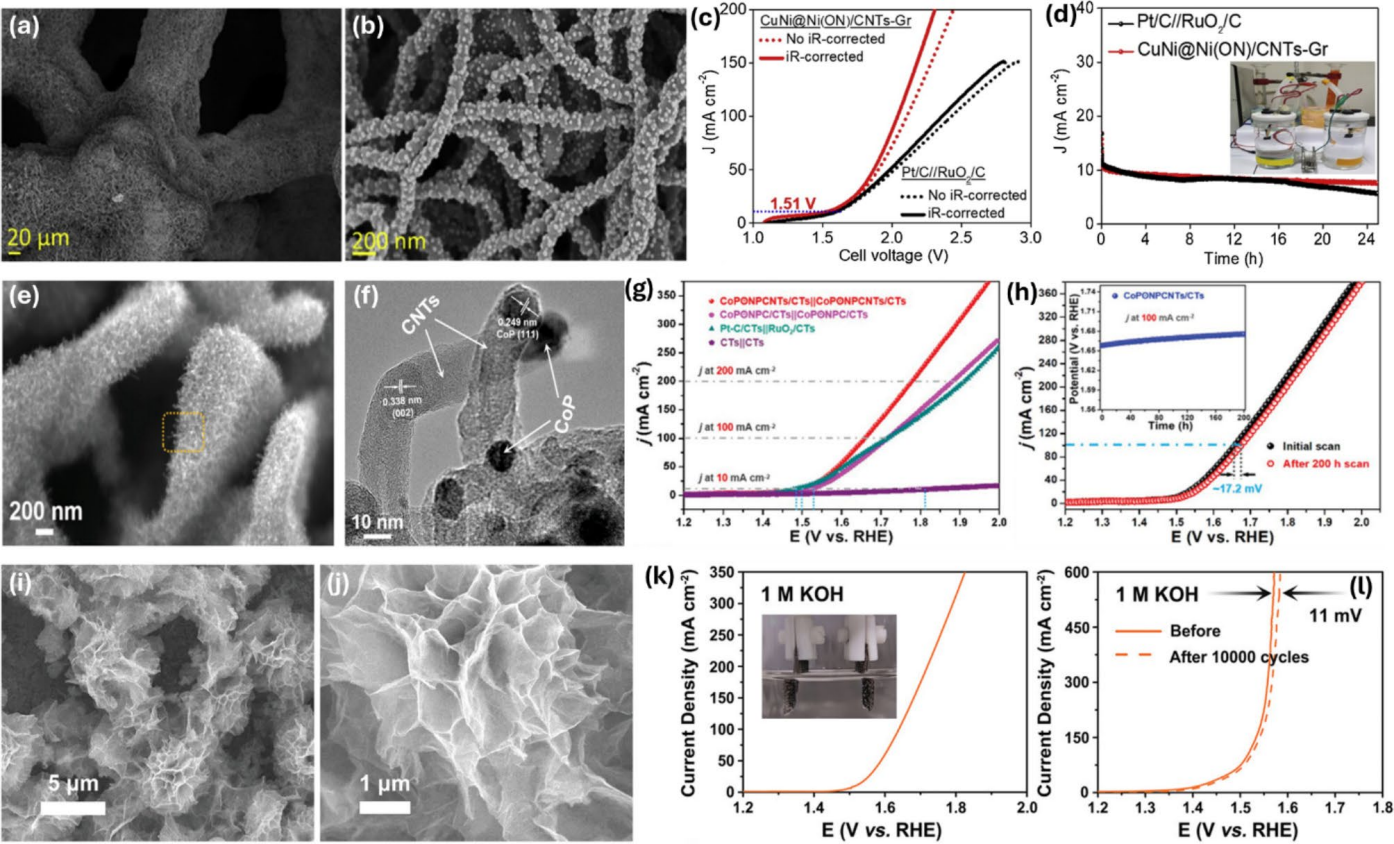
(**a**-**b**) Structure of the CuNi@Ni(ON)/CNTs-Gr heterostructure and (**c**-**d**) OWS performance of the CuNi@Ni(ON)/CNTs-Gr_(+,-)_ couple. Images reproduced from Ref [114]. with permission from Wiley; (**e**-**f**) Structure of the catalyst based on CoP NPs embedded into N, P-codoped CNTs and (**g**-**h**) its OWS performance. Images reproduced from Ref [115]. with permission from Elsevier; (**i**-**j**) Structure of leaf-like Co_2_P-NC and (**k**-**l**) OWS performance of the Co_2_P-NC_(+,-)_ couple. Images reproduced from Ref [117]. with permission from Wiley

The production of hydrogen via electrochemical water splitting, consisting of two half reactions, OER and HER, often requires high-performance anodes and cathodes to not only reduce the η of the reactions but also enhance the stability and durability of the electrolyzer. Currently, commercial electrodes are categorized into two types: active powder-deposited on conductive supporting matrices and self-supported electromaterials. Unfortunately, electrodes based on powder catalysts require nonconductive binders (e.g., Nafion), which obstruct the exposure of active sites, charge transfer, and mass transport [104]. Additionally, powder catalysts can be dislodged by gas bubbles at high applied current density during operation. Conversely, self-supported electrocatalysts offer significant advantages, addressing these issues [105–107], such as: (i) direct anchoring of more active materials with abundant active sites on the supporting substrate during an in-situ synthesis process; (ii) no use of binder, thereby preventing inhibition of active sites; (iii) fabrication of an appropriate 3D architecture with a large active surface area and reasonable porosity to enable fast charge and mass transfer; (iv) strong interactions between active catalysts and substrate protect the catalysts from leaching during OWS at high current densities. Therefore, self-supported electrocatalysts have recently emerged as promising candidates for practical OWS applications. Numerous studies are focusing on developing self-supported electrocatalysts using various methods, such as solvothermal/hydrothermal [108, 109], electrodeposition [110–112], and chemical deposition [113]. These 3D open-architecture substrates, such as NF, copper foam, or carbon cloth, have shown great potential with high structural stability and catalytic activity. Specifically, our group recently demonstrated the synthesis of a unique nanohybrid, CuNi@Ni core@shell NPs dual-coordinated with O and N on 3D porous CNTs-GR through effective electrodeposition and thermal treatment processes [114]. The CuNi@Ni(ON)/CNTs-Gr material, consisting of CuNi@Ni(ON) NPs with diameters of approximately 20–30 nm, is uniformly attached to CNTs without aggregation (Fig. 8a-b). This structure exhibits tunable electronic properties and conductivity that significantly promote bifunctional activities towards HER and OER in 1.0 M KOH. An electrolyzer equipped with CuNi@Ni(ON)/CNTs-Gr electrodes achieves a low cell voltage of 1.51 V at 10 mA cm^− 2^ and exhibits robust durability over a 25-hour operation, outperforming the Pt/C//RuO_2_ pair (Fig. 8c-d). Kong et al. also introduced a unique 3D hierarchical nanostructure consisting of ultra-small CoP NPs embedded into N, P-codoped CNTs knitted into hollow nano-wall arrays on carbon textiles using a carbonization-phosphidization process [115]. The CoP NPs are well supported and shielded by conductive N, P-codoped CNTs knitted into hollow nano-wall arrays, providing abundant active sites and favorable diffusion pathways (Fig. 8e-f). Owing to the uniform distribution of CoP NPs and high porosity, the material demonstrates superior HER and OER activity and stability in alkaline medium. The assembled electrolyzer delivers impressive performance with a low cell voltage of 1.50 V at 10 mA cm^− 2^ for OWS, supplemented by advantageous stability (Fig. 8g-h). In another study, Mu et al. developed a superwetting metal selenide-based electrode from a peony-flower-shaped micro-nano array of MoS_2_/Co_0.8_Fe_0.2_Se_2_/Ni_*x*_Se_*y*_ on NF using a two-step hydrothermal method [116]. This electrode, characterized by a unique change in electronic properties, facilitates electrolyte penetration, mass transfer, and bubble detachment due to its superwetting properties, leading to superior OER and HER performance, eventually enhancing OWS in a 1.0 M KOH solution.

Wang et al. recently described the creation of a high-performance, cost-effective, and rapidly produced bifunctional material based on a novel self-supported structure of Co-doped ammonium lanthanum molybdate with stacked nanosheets featuring abundant crystalline-noncrystalline boundaries on NF (Co-ALMO@NF) (Fig. 8i-j) [117]. This material possesses a large active surface, numerous defects, and multiple-conductive channels that elevate the electrocatalytic performance of HER and OER, thereby requiring only a voltage of 1.52 V at 10 mA cm^− 2^, along with excellent long-term stability (Fig. 8k-l**).** The status of self-supported electrocatalyst development for OER, HER, or bifunctional OER & HER is a highly active area currently, with numerous research groups participating. Consequently, a variety of self-supported electrocatalyst candidates with diverse morphologies, structures, and chemical compositions are under investigation, fabrication, and application for practical commercialization towards OWS [118–122].

### Doping effect-based electrocatalysts

**Fig. 9:**
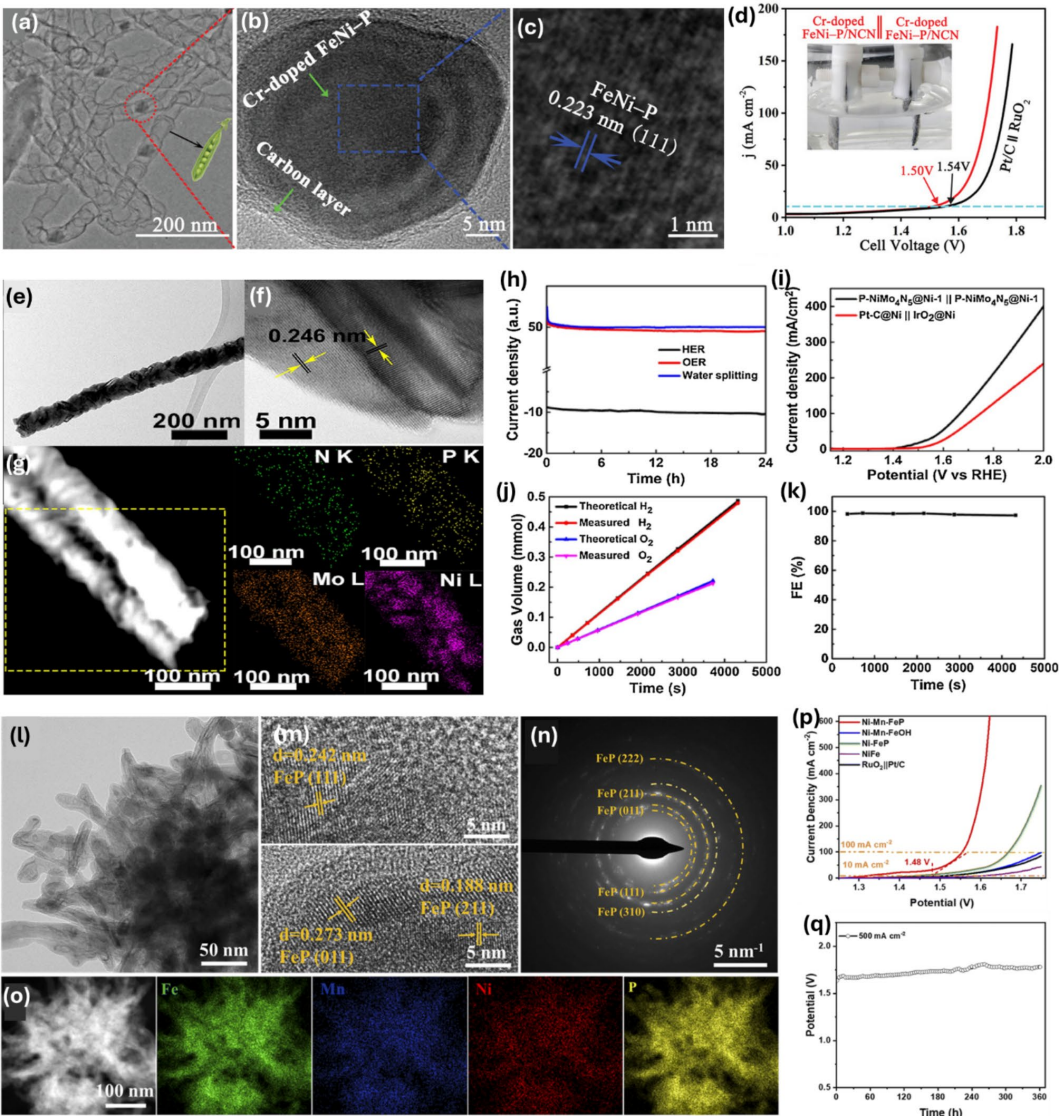
(**a**-**c**) Structure of Cr-doped FeNi–P/NCN hybrid and (**d**) OWS performance of the FeCo-P_(+,-)_ couple. Images reproduced from Ref [128]. with permission from Wiley; (**e**-**f**) Structure of the hierarchical P-NiMo_4_N_5_@Ni catalyst and (**g**-**h**) OWS performance of the P-NiMo_4_N_5_@Ni_(+,-)_ couple. Images reproduced from Ref [129]. with permission from Elsevier; (**j**-**n**) Structure of leaf-like Co_2_P-NC and (**o**-**p**) OWS performance of the Co_2_P-NC_(+,-)_ couple. Images reproduced from Ref [130]. with permission from the RSC

From a structural engineering perspective, in addition to the development of SACs, core@shell heterostructures, and self-supported materials, enhancing catalytic properties towards HER and OER in water electrolysis is achieved by employing metallic (Fe, Pt, Co, Ru, Mn, Pd, Ni, Ru, W, Mo, Cu…) or non-metallic (P, S, B, N, Se…) doping strategies. These strategies are promising for optimizing adsorption free energy towards reactants/intermediates, exposing more active sites, increasing conductivity, and improving selectivity for catalytic reactions. Recent advances in doping strategy have offered insights into the relationship between doping effects and catalytic properties, facilitating the rational design of high-performance catalysts for OWS. For example, Ghosh et al. obtained a catalyst composed of 15% W-Ni_12_P_5_, which exhibits high catalytic activity in 1.0 M KOH with η of 172 mV for HER and 322 mV for OER at 10 mA cm^–2^ [123]. The authors identified that the formation of an amorphous surface layer of WO_*x*_-Ni_*x*_P at higher doping content significantly promotes the reactions. Recent studies have confirmed that doping elements such as Ni, Co, Fe, and O can effectively modify the electronic structure of MoS_2_ and adjust the charge density of Mo to lower the hydrogen adsorption free energy, resulting in enhanced catalytic performance for HER [124–127]. For example, Xiong discovered that doping layer-structured MoS_2_ with Co led to significant enhancements in HER and OER activities, achieving low η of 48 and 260 mV at 10 mA cm^− 2^, respectively [127]. Moreover, Wu et al. developed a Cr-doped FeNi–P/NCN hybrid using a one-step heating method, capturing small Cr-doped FeNi–P NPs with a diameter of 35 nm within a carbon layer (Fig. 9a-c) [128]. DFT calculations suggest that Cr doping within the FeNi structure synergistically adjusts the adsorption energy, enhances charge transfer, and boosts electrocatalytic activity. The Cr-doped FeNi–P/NCN displays exceptional OER and HER performance, achieving η of 240 and 190 mV, respectively, to reach a current response of 10 mA cm^− 2^ in 1 M KOH medium. Consequently, the OWS enabled by this bifunctional catalyst requires only 1.50 V to attain 10 mA cm^− 2^, outperforming the Pt/C//RuO_2_ couple, which requires 1.54 V (Fig. 9d).

Recently, Shen et al. introduced a finely controlled design for the synthesis of various morphological arrays using hierarchical P-NiMo_4_N_5_@Ni catalysts on NF (Fig. 9e-g) [129]. The optimized electrode of P-NiMo_4_N_5_@Ni, boasting a large surface-to-volume ratio of active sites, demonstrates outstanding activity for OWS, achieving a current density of 50 mA cm^− 2^ at a low cell voltage of 1.59 V, long-term stability, and high faradaic efficiency (Fig. 9h-k). Of special interest, a multi-doping approach with two or more heteroatoms has been shown to further enhance the electrocatalytic performance through synergistic effects [131–133]. For instance, Liu et al. reported modifying the d-band centers in FeP by dual metal doping with Ni and Mn for OWS [130]. The FeP nanoarrays, directly grown on NiFe foam with integrated Ni and Mn dual-dopants (Ni–Mn–FeP), were synthesized through etching-depositing and phosphidization routes (Fig. 9l-o). Theoretical studies indicate that this modification of the d-band center leads to high intrinsic activity with notable enhancements in *O-*OOH conversion and H* adsorption. Consequently, this material requires only an η of 185 mV for OER and 103 mV for HER to achieve 10 mA cm^− 2^ in 1.0 M KOH. The bifunctional Ni–Mn–FeP cathode and anode in an electrolytic cell can produce a current of 100 mA cm^− 2^ at a low cell voltage of 1.55 V with stability over 360 h at 0.5 A cm^− 2^, suggesting great potential for large-scale OWS applications (Fig. 9p-q). Despite the various ongoing research efforts into doping strategies for catalyst development, a deeper understanding of the effects of heteroatom-doping, and the unique properties of the resulting electrocatalysts influenced by functional groups, electronic structure, atomic size, dopant type, and the location and distribution of active sites, is required to enhance their catalytic performance. There remain numerous challenges in developing the next generation of highly efficient electrocatalysts to meet the demands of commercial WOS applications.

## Current development status of industrial electrocatalysts and electrolyzers for OWS

**Fig. 10:**
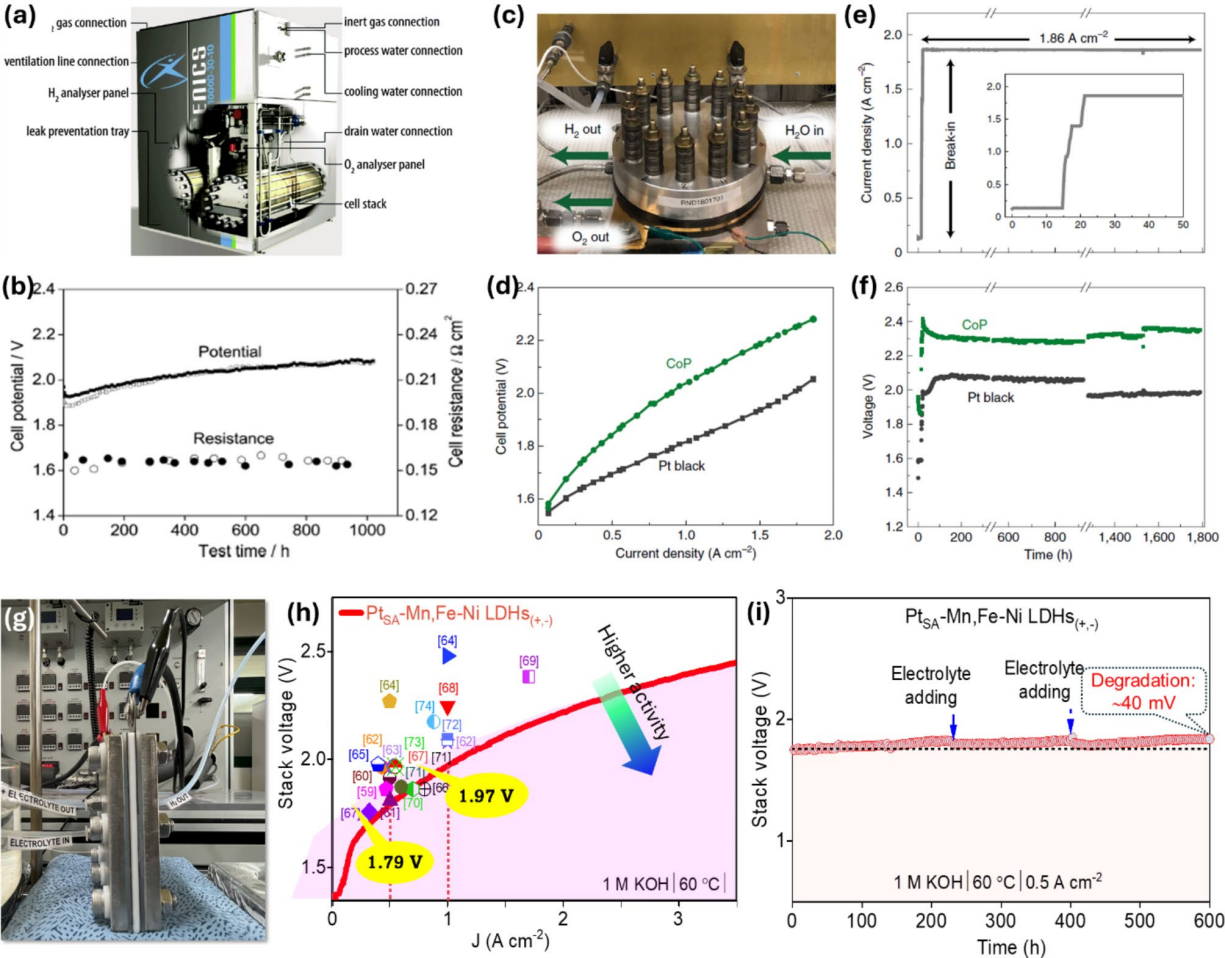
(**a**) A commercially available AWE manufactured by Hydrogenics Co., complete with auxiliary components. Images reproduced from Ref [137]. with permission from RSC; (**b**) Long-term performance and AC resistance (1 kHz) of AEMWE cells with a HER catalyst of 7.4 mg cm^-2^ operating at 3 MPa, at a current density of 0.47 A cm^-2^, and 316 K in electrolyte solutions consisting of 1 wt% K_2_CO_3_/KHCO_3_ (solid symbol) and 1 wt% K_2_CO_3_ (open symbols). Image reproduced from Ref [138]. with permission from Willey; (**c**-**f**) PEMWE developed by Nel Hydrogen, accompanied by its performance tests. Image reproduced from Ref [141]. with permission from Nature; (**g**-**i**) AEMWE developed by AHES Co., alongside its performance tests. Image reproduced from Ref [43]. with permission from ACS

The PEMWE, AWE, and AEMWE are emerging as promising low-temperature H_2_ production technologies [134], with ongoing optimization efforts aimed at achieving commercialization targets for future electrochemical hydrogen production. Currently, research focused on enhancing the efficiency of electrolyzer devices falls into three primary categories: (i) novel cell configurations, including zero-gap designs and low-resistance diaphragms, (ii) high-pressure operations, and (iii) new electrocatalysts. Regarding catalyst and electrolyzer development, extensive research into high-performance commercial OWS systems is underway at various manufacturers in developed countries such as Tianjin Mainland Hydrogen (China), Hydrogenics (Canada), Elchemtech (Korea), AHES (Korea), Proton Onsite Co. (USA), Siemens (Germany), Kobelco (Japan), Giner (USA), Teledyne (USA), 3 M (USA), ITM Power (UK), ArevaH2Gen (France), and others [135]. The AWE demonstrates a low H_2_ production rate of 200 mA cm^− 2^ at a cell voltage of 1.8 V, with an energy efficiency of 75%_HHV_ [3]. In comparison, the AEMWE can produce H_2_ at approximately ∼1.8 A cm^− 2^ at 2 V, nearing PEMWE’s conventional performance. A current zero-gap design of PEMWEs allows operation at roughly∼2 A cm^− 2^ with an efficiency of 74%_HHV_ [3]. However, based on the recent development trend in [4], the research starting at an earlier period for AWE and AEMWE is more established and mature compared to PEMWE, due to the lower durability and expensive materials mandatory for PEMWE technology [3]. Additionally, global potential funding is demonstrating increased interest in hydrogen production and electrolyzers. Hydrogenics Co., a relatively new yet prominent company, is quickly becoming a key player in the global production of electrocatalysts and electrolyzers for safe and reliable production of green and pure H_2_ [136]. Hydrogenics offers two electrolysis technologies: AWE and PEMWE. Figure 10a shows a commercially available AWE, the HySTATt, from Hydrogenics [137]. As a pioneer in the green H_2_ production market, Acta S.p.A. (ACTA) Co. provides AEMWE systems capable of producing 100–1000 L H_2_/h at 3 MPa. To enhance the efficiency of water electrolyzers, this company also developed cost-effective, non-precious commercial electrocatalysts suitable for alkaline conditions [138]. Two types of commercial catalysts, Acta 4030 (Ni/CeO_2_-La_2_O_3_/C) and Acta 3030 (CuCoO_x_), were evaluated as HER and OER catalysts for AEMWE, respectively, in mildly alkaline environments (pH 10–11). The cell voltage was reduced from 2.01 to 1.89 V at a current density of 470 mA cm^-2^ due to an increase in the amount of HER catalyst from 0.6 to 7.4 mg cm^-2^ (Fig. 10b) [8, 138]. The cell potential remained unchanged from 800 h to the end of the test. Johnson Matthey Co. (UK) designs commercial high-performance catalysts based on Raney Nickel, which exhibit enhanced HER activity and stability in alkaline media, comparable to catalysts based on the Pt metal group. The performance of Mo-doped Raney Nickel-based catalysts was tested for commercial electrolyzers in 38% KOH (pH = 15.15 at 25 °C). The developed AWE (10 cm^2^) with Raney Ni-Mo cathode, Raney Ni-Fe anode, and Celgard separators performed better than commercial PEMWE at current densities below ∼300 mA cm^-2^ [139]. Proton OnSite (USA), a major manufacturer in the field, focuses on fabricating and supplying AWE and PEMWE devices, capable of producing 400,000 L H_2_ h^-1^ at 3 MPa. The company recently developed a successful non-Pt group metals (PGM) catalyst made of cubic LiCoO_2_ (or LiCo_2_O_4_), which serves as an effective OER catalyst compared to superior PGM catalysts in AEMWE [33]. The fabricated AEMWE, utilizing a LiCoO_2_ anode and Pt cathode, operates at a required cell voltage of 1.91 V at a current density of 400 mA cm^-2^ and remains stable for over 1000 h at 50 ^o^C [140]. In China, AWEs have been extensively commercialized by large domestic producers such as Beijing CEI Technology Co. and Tianjin Mainland Hydrogen Equipment Co., Ltd. (THE), recognized as a leading global supplier of alkaline electrolyzers with more than 400 production plants since 1994.

Cockerill JingLi Hydrogen Co., renowned for its focus on R&D and the production of electrolyzers, can produce H_2_ capacities ranging from 2 m³ h^-1^ to 1500 m³ h^-1^ and is exported to over 30 countries globally. Thyssenkrupp recently introduced industrial-scale water electrolysis for large projects with efficiencies of over 80% that convert OWS into H_2_ and O_2_. The Nel Hydrogen Company, a well-known industry player in AWE and PEMWE systems, can generate hydrogen at rates up to 3,880 Nm^3^ h^-1^, equivalent to just over 8 tons per day, suitable for various applications. Particularly, their M Series electrolyzers can produce H_2_ gas up to 4,000 Nm^3^ h^-1^ at 99.9998% purity on-demand. To date, this company has delivered electrolyzer solutions totaling over 3500 to more than 80 countries [142]. Instead of using a Pt catalyst at the cathodic side, the Nel Hydrogen team has experimented with low-cost powder alternatives based on cobalt phosphide NPs-deposited carbon substrate, achieving stable current density at 1.86 A cm^− 2^ during 1,763 h of testing (Fig. 10c-f) [3, 141]. Collaborative work by AHES Co. in Korea has developed an anion exchange membrane electrolyzer stack using Pt_SA_–Mn, Fe–Ni LDHs_(+,−)_ that requires stack voltages of 1.79 and 1.97 V at 0.5 and 1.0 A·cm^–2^, respectively, demonstrating outstanding durability over 600 h and showing high potential for commercial OWS applications (Fig. 10g-i) [43]. An overview of recent catalyst and electrolyzer advancements in commercial PEMWE, AWE, AEMWE systems is presented in detail below. Table 1 displays the performance data of recent commercial PEMWE and AWE systems developed by various companies [135].

**Table 1:** Recent developments in electrolyzers and their performance data as reported by various manufacturers [135]

Manufacturer	Technology	Production rate (Nm^3^ /h)	Power (kW)	Energy use (kWh/Nm^3^)	Efficiency^a^	Max, pressure (bar)	H_2_ purity (vol%)	Location
AccaGen	Alkaline (bipolar)	1-100	6.7–487	6.7-4.87^b^	52.8–72.7	10 (optional 30) (200^c^)	99.9 (99.999^d^)	Switzerland
A valence	Alkaline (monopolar)	0.4–4.6 (139^c^)	2–25 (750^c^)	5.43-5	65.2–70.8	448	n.a.	USA
ELT	Alkaline (bipolar)	3-330	13.8–1518	4.6–4.3	76.9–82.3	Atm.	99.8–99.9	Germany
ELT	Alkaline (bipolar)	100–760	465–3584	4.65–4.3	76.1–82.3	30	99.8–99.9	Germany
Erredue	Alkaline (bipolar)	0.6–21.3	3.6–108	6.5-1^b^	59-69.8	2.5-4	99.3–99.8 (99.999^d^)	Italy
Giner	PEM (bipolar)	3.7	20	5.4^e^	65.5	85	n.a.	USA
Hydrogen Technologies, Division of Statoil	Alkaline (bipolar)	10–500	43-2150	4.3	82.3	Atm.	99.9	Norway
Hydrogenics	Alkaline (bipolar)	10–60	54–312	5.4-5.2^b^	65.5–68.1	10 (optional 25)	99.9 (99.999^d^)	Canada
Hydrogenics	PEM (bipolar)	1	7.2	7.2^b^	49.2	7.9	99.99	Canada
H_2_ Logic	Alkaline (bipolar)	0.66–42.62	3.6–213	5.45-5^b^	64.9–70.8	4 (optional 12)	99.3–99.8 (99.999^d^)	Denmark
Idroenergy	Alkaline (bipolar)	0.4–80	3-377	7.5–4.71	47.2–75.2	1.8-8	99.5	Italy
Industrie Haute Technologie	Alkaline (bipolar)	110–760	511.5–3534	4.65–4.3	76.1–82.3	32	99.8–99.9	Switzerland
Linde	Alkaline (bipolar)	5-250	n.a.	n.a.	n.a.	25	99.9 (99.998^d^)	Germany
PIEL, division of ILT Technology	Alkaline (bipolar)	0.4–16	2.8–80	7.5^b^	50.6–70.8	1.8–18	99.5	Italy
Proton OnSite	PEM (bipolar)	0.265-30	1.8–174	7.3–5.8	48.5–61	13.8–15 (optional 30)	99.999	USA
Sagim	Alkaline (monopolar)	1–5	5–25	5	70.8	10	99.999	France
Teledyne Energy	Alkaline (bipolar)	2.8–56	n.a.	n.a.	n.a.	10	99.999	USA
System Treadwell Corporation	PEM (bipolar)	1.2–10.2	n.a.	n.a.	n.a.	75.7	n.a.	USA

*n.a*: information not available; *a*: Calculated based on the HHV of hydrogen (3.54 kWh/Nm^3^); *b*: Based on the global hydrogen production industry; *c*: Currently in development; *d*: Includes an additional purification system; *e*: Solely based on the electrolysis process.

## Conclusions and outlook

Given the significant impact of human activities on climate change, there is a swift global shift from fossil fuels to advanced, sustainable, renewable, clean, and decarbonized energy sources. In this context, electrochemical water splitting has emerged as a viable green technique for clean H_2_ production. Developing high-performance electrocatalysts along with efficient electrolyzer configurations and devices is crucial for achieving major technological progress. In recent years, ongoing research and development efforts in academic institutions, universities, and company R&D centers in the fields of electrocatalysts and electrolyzers have aimed to enhance performance, efficiency, and cost-effectiveness, thereby boosting their competitiveness and viability for a broad range of future applications. Although recent achievements have marked considerable progress in practical applications, water splitting technology still involves significant technical risks, necessitating ongoing research to meet industrialization demands. Key research areas for the community include: (i) Developing advanced catalysts for both HER and OER, which are essential for bolstering the overall efficiency of electrolyzer technology; (ii) Discovering new materials for components of electrolyzers, like electrolyte membranes and bipolar plates, to improve electrical conductivity and chemical stability, mitigate corrosion, and enhance efficiency and durability; (iii) Improving system integration techniques, such as stack configuration design, thermal management, and control strategies, which help reduce energy losses, increase system reliability, and lower overall costs. On the other hand, the structure-activity relationship can be also investigated by theoretical calculations to better understand the atomic insight on HER and OER processes. Successfully optimizing these engineering strategies (Table 2) is vital for advancing electrolyzer technology in the rapidly expanding global market, expected to surpass $45.48 billion by 2032, with a CAGR of ∼32.2% from now until 2032 [143].

**Table 2:** Overview of key performance indicators for recent and future PEMWE, AWE, and AEMWE technologies [144]

	2020	Target 2050	*R*&D focus
	PEMWE		
Nominal current density (A cm^− 2^)	1–2	4–6	Design, membrane
Voltage range (limits) (V)	1.4–2.5	< 1.7	Catalyst, membrane
Operating temperature (°C)	50–80	80	Effect on durability
Cell pressure (bar)	< 30	> 70	Membrane, reconversion catalysts
Load range (%)	5-120	5-300	5-300% Membrane
H_2_ purity (%)	99.9-99.9999	Same	Membrane
Voltage efficiency (LHV) (%)	50–68	> 80	Catalysts
Electrical efficiency (stack) (kWh/Kg H_2_)	47–66	< 42	Catalysts/membrane
Electrical efficiency (system) (kWh/Kg H_2_)	50–83	< 45	Balance of plant
Lifetime (stack) (h)	50 000–80 000	100 000-120 000	Membrane, catalysts, PTLs
Stack unit size (MW)	1	10	MEA, PTL
Electrode area (cm²)	1 500	> 10 000	MEA, PTL
Cold start (to nominal load) (min)	< 20	< 5	Insulation (design)
Capital Costs (stack) minimum 1 MW (USD/kW)	400	< 100	MEA, PTLs, BPs
Capital Costs (system) minimum 10 MW (USD/kW)	700–1400	< 200	Rectifier, water purification
	**AWE**		
Nominal current density (A cm^− 2^)	0.2–0.8	> 2	Diaphragm
Voltage range (limits) (V)	1.4-3	< 1.7	Catalysts
Operating temperature (°C)	70–90	> 90	Diaphragm, frames, balance of plant components
Cell pressure (bar)	< 30	> 70	Diaphragm, cell, frames
Load range (%)	15–100	5-300	Diaphragm
H_2_ purity (%)	99.9-99.9998	> 99.9999	Diaphragm
Voltage efficiency (LHV) (%)	50–68	> 70	Catalysts, temperature
Electrical efficiency (stack) (kWh/Kg H_2_)	47–66	< 42	Diaphragm, catalysts
Electrical efficiency (system) (kWh/Kg H_2_)	50–78	< 45	Balance of plant
Lifetime (stack) (h)	60 000	100 000	Electrodes
Stack unit size (MW)	1	10	Electrodes
Electrode area (cm²)	10 000–30 000	30 000	Electrodes
Cold start (to nominal load) (min)	< 50	< 30	Insulation (design)
Capital Costs (stack) minimum 1 MW (USD/kW)	270	< 100	Electrodes
Capital Costs (system) minimum 10 MW (USD/kW)	500-1 000	< 200	Balance of plant
	**AEMWE**		
Nominal current density (A cm^− 2^)	0.2-2	> 2	Membrane, reconversion catalysts
Voltage range (limits) (V)	1.4-2.0	< 2	Catalyst
Operating temperature (°C)	40–60	80	Effect on durability
Cell pressure (bar)	< 35	> 70	Membrane
Load range (%)	5-100	5-200	Membrane
H_2_ purity (%)	99.9-99.999	> 99.9999	Membrane
Voltage efficiency (LHV) (%)	52–67	> 75	Catalysts
Electrical efficiency (stack) (kWh/Kg H_2_)	51.5–66	< 42	Catalysts/membrane
Electrical efficiency (system) (kWh/Kg H_2_)	57–69	< 45	Balance of plant
Lifetime (stack) (h)	> 5 000	100 000	Membrane, electrodes
Stack unit size (MW)	0.0025	2	MEA
Electrode area (cm²)	< 300	1 000	MEA
Cold start (to nominal load) (min)	< 20	< 5	Insulation (design)
Capital Costs (stack) minimum 1 MW (USD/kW)	Unknown	< 100	MEA
Capital Costs (system) minimum 10 MW (USD/kW)	Unknown	< 200	Rectifier

## Data Availability

The review paper content is based on the reusing option of published data in the reference list enabled by Copyright Clearance Center’s RightsLink service.
